# Infectious Causes of Acute Febrile Illness in Belize, 2020–2022

**DOI:** 10.4269/ajtmh.25-0328

**Published:** 2025-12-04

**Authors:** Adrianna Maliga, Shannon E. Ronca, Russell Manzanero, Roberto Melendez, Sarah M. Gunter, Allison Lino, Joy Mackey, Andres Espinosa-Bode, Beatriz Lopez, Rafael Chacon-Fuentes, Freedom M. Green, Oluwadara Okeremi, Sarah Strobel, Daniel P. Rehm, Flor M. Muñoz, Loren Cadena, Melissa Diaz-Musa, Francis Morey, Emily Zielinski-Gutierrez, Gerhaldine Morazan, Kristy O. Murray

**Affiliations:** ^1^ Department of Pediatrics, Baylor College of Medicine and Texas Children’s Hospital, Houston, Texas;; ^2^ William T. Shearer Center for Human Immunobiology, Texas Children’s Hospital, Houston, Texas;; ^3^ Belize Ministry of Health and Wellness, Belmopan, Belize;; ^4^ Henry J.N. Taub Department of Emergency Medicine, Baylor College of Medicine, Houston, Texas;; ^5^ Centers for Disease Control and Prevention—Central America, Guatemala City, Guatemala;; ^6^ Department of Pediatrics, Emory University School of Medicine and Children’s Healthcare of Atlanta, Atlanta, Georgia

## Abstract

Acute febrile illness (AFI) surveillance networks can play a key role in identifying emerging pathogens and promoting global health security, especially for vulnerable populations in low- and middle-income countries. In January 2020, the Belize AFI Surveillance network was formed, and 5,643 participants were enrolled over the first 3-year period across 11 participating public hospitals. Using real-time polymerase chain reaction testing for vector-borne pathogen detection and BioFire® diagnostic testing for respiratory and gastrointestinal pathogens, the causes of illness were examined in relation to 54 pathogens in these participants. In response to the coronavirus disease 2019 pandemic, surveillance was expanded to detect severe acute respiratory syndrome coronavirus 2 (SARS-CoV-2) infections. Overall, one or more pathogens were successfully identified in 51% of participants, with 43 different pathogens detected. Many important discoveries were made, including the first reported case of acute Chagas disease in Belize and eight cases of *Vibrio cholerae*. The most common identified vector-borne disease was dengue, with all four serotypes detected. The most common respiratory pathogens detected were SARS-CoV-2 and human rhinovirus/enterovirus. The most common gastrointestinal pathogens detected were different strains of diarrheagenic *Escherichia coli* and norovirus. These and other results obtained via AFI surveillance enabled in-country and academic partners to respond to disease outbreaks rapidly and to monitor disease activity across the country more effectively.

## INTRODUCTION

Emerging and reemerging pathogens are global public health threats. Countries in Central America face disproportionately high risks for transmission because of large population centers, areas of extreme poverty, limited resources for surveillance and diagnostics, and tropical climates.^1^ In the Central American country of Belize, 41% of its ∼400,000 residents live in poverty,^2^ increasing the risk of infections and poor health outcomes. Additionally, with ∼1 million tourist visits each year, the risk of introducing and spreading vector-borne, respiratory, and enteric pathogens, both to and from the country, is amplified.^3^

Mosquito-borne viruses are common in Belize, especially during the rainy season (June to December). Dengue virus (DENV) outbreaks have been reported almost annually since the 1980s,^4^ and the first locally acquired cases of chikungunya virus (CHIKV) and Zika virus (ZIKV) were reported in 2014 and 2015, respectively.^5^^–^^8^ With *Aedes aegypti* mosquito populations established throughout the region and limited resources for vector surveillance and control, pathogen transmission continues. West Nile virus (WNV) has been detected in birds in Central America, but no human cases have yet been identified.^9^^,^^10^ The autochthonous transmission of parasitic and bacterial vector-borne diseases (VBDs) also occurs; however, the true incidence and prevalence of these infections are not well documented because of limited diagnostics.^11^

In addition to VBDs, respiratory pathogens pose a significant global health threat, particularly in children. In 2008, Nair et al. estimated that 28,000 to 111,500 deaths in children <5 years of age were attributable to influenza-associated acute lower respiratory tract illness, with 99% of these deaths occurring in low- and middle-income countries (LMICs).^12^ Similarly, in 2015, 33.1 million respiratory syncytial virus (RSV) infections occurred in children in LMICs, in contrast to the 2.8 million cases that occurred in high-income countries.^12^^,^^13^ Infection with enteric pathogens also contributed to morbidity and mortality, with poor sanitation and high population densities in urban settings influencing ongoing transmission.^14^ Additionally, up to 50% of visitors to Central America (for 2 weeks or longer) are estimated to develop enteric infections, with foodborne transmission being a primary contributor.^15^^,^^16^ There is a clear need for enhanced surveillance and improved public health infrastructure to detect, report, and respond to infectious diseases.

Acute febrile illness (AFI) surveillance networks can play a key role in identifying emerging pathogens and promoting global health security, especially for vulnerable populations in LMICs.^17^ To better detect, characterize, and define these disease threats in Belize, Baylor College of Medicine (BCM), along with the Ministry of Health and Wellness (MOHW) of Belize and the US CDC, created a country-wide, multi-disciplinary AFI surveillance network. Vector-borne, respiratory, and enteric pathogens were monitored in hospitals and outpatient clinics (polyclinics) across the country to detect and respond to infectious disease threats. The study surveillance findings from 2020 to 2022, including the integration of severe acute respiratory syndrome coronavirus 2 (SARS-CoV-2) into emergency response surveillance at the onset of the pandemic, are presented in this work.

## MATERIALS AND METHODS

### Surveillance sites.

**Figure 1.**
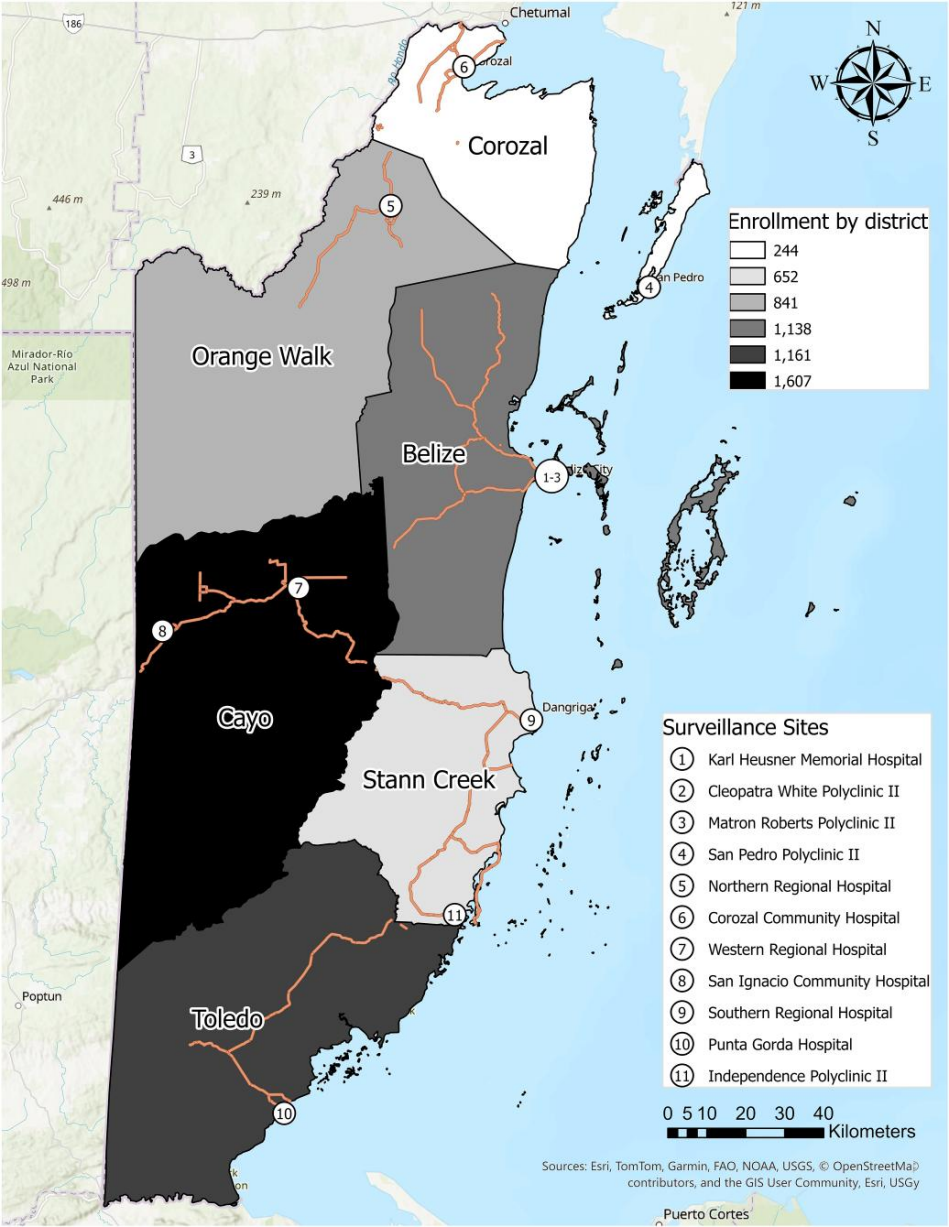
Map of enrollment sites. Pins indicate locations of the healthcare facilities participating in the acute febrile illness surveillance network.

The Belize AFI surveillance network enrolled participants at all 11 MOHW-supported regional hospitals and polyclinics throughout the country. Each had established transportation networks for specimen transport to the MOHW Central Medical Laboratory in Belize City (Figure 1). Clinical sites include the Karl Heusner Memorial Hospital Authority, Matron Roberts Polyclinic II, Cleopatra White Polyclinic II, San Pedro Polyclinic II, and Dr. Otto Rodriguez in Belize District; the Corozal Community Hospital and Polyclinic in Corozal District; the Northern Regional Hospital and Polyclinic in Orange Walk District; the Western Regional Hospital and Polyclinic and San Ignacio Community Hospital in Cayo District; the Southern Regional Hospital and Polyclinic and Independence Polyclinic II in Stann Creek District; and the Punta Gorda Community Hospital and Polyclinic in Toledo District (Figure 1).

### Inclusion criteria.

Beginning January 16, 2020, patients presenting to participating sites were invited to enroll as study participants if their axillary, oral, or rectal temperature met or exceeded 38°C or if they presented with a history of new-onset objective or subjective fever within the past 7 days. All participants who were at least 60 days old were eligible for inclusion.

**Figure 2.**
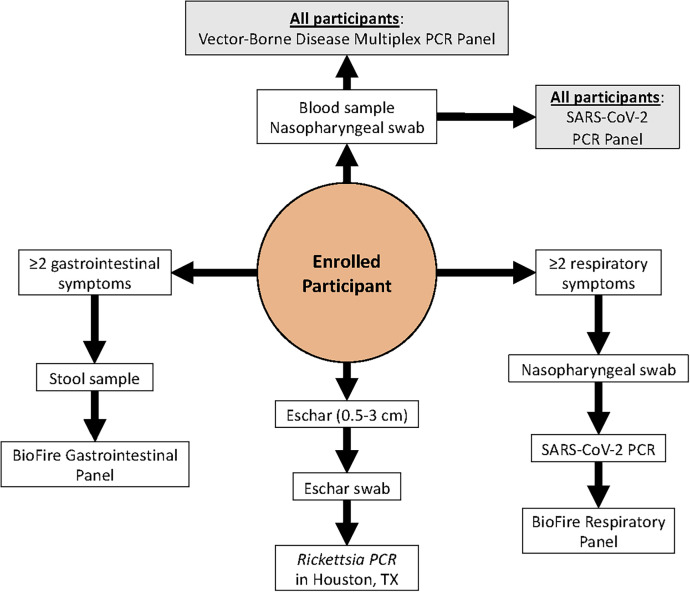
Participant testing algorithm based on clinical presentation for the acute febrile illness study. All enrolled participants received testings against the vector-borne disease panels, as well as additional testing related to symptoms at enrollment. Beginning August 19, 2020, all participants underwent severe acute respiratory syndrome coronavirus 2 testing using samples from a collected nasopharyngeal swab, regardless of respiratory symptoms.

In response to the SARS-CoV-2 pandemic, on March 17, 2020, the inclusion criteria were expanded to include nonfebrile patients presenting with two or more respiratory symptoms, including cough, loss of taste or smell, sore throat, painful swallowing, wheezing, chest pain, sneezing, shortness of breath, runny nose, and nasal congestion. On September 29, 2020, the criteria were further expanded to include nonfebrile patients presenting with two or more gastrointestinal (GI) symptoms, including diarrhea, nausea, vomiting, abdominal or stomach pain, upset stomach, black stool, and blood in stool, as well as those considered high risk for SARS-CoV-2 infection because of a known exposure, occupation as a healthcare worker, residence in a nursing home or prison, recent travel to an endemic area, or atypical clinical presentation suspected to be caused by SARS-CoV-2 (Figure 2).

### Exclusion criteria.

Participants were excluded from the study if they 1) did not or were unable to provide consent or assent; 2) did not consent to follow-up 2 to 3 weeks after enrollment; 3) had been previously enrolled in the AFI surveillance study within the past 7 days or returned with an unresolved illness related to prior enrollment; 4) returned for the continuation of treatment of a known cause of fever; or 5) presented with a chief complaint of injury or trauma, a known cause of fever (nonenteric, non-respiratory, non-vector-borne), or other clear source of infection (i.e., abscess) when examined by a healthcare provider.

### Enrollment procedures.

Written informed consent was obtained from all participants or their legal representative if the participant was unable to provide consent because of a medical condition or was under 9 years of age. For children aged 9 to 18 years, written assent was obtained in addition to parental or guardian permission. Participants were interviewed at the time of enrollment to capture demographic data (date of birth, sex, pregnancy status, race/ethnicity, and occupation), epidemiological information (travel history, exposure to ill individuals, and vector and animal exposure history), and clinical data (general, respiratory, and GI symptoms and coronavirus disease 2019 [COVID-19] vaccine history).

### Sample collection.

Whole blood (WB; up to 3 mL collected in Ethylenediaminetetraacetic acid) and serum (up to 3 mL) were collected on all participants (Figure 2). From January 16, 2020 through August 19, 2020, nasopharyngeal (NP) swabs were only collected from those presenting with two or more respiratory symptoms. After August 19, 2020, NP swabs were collected from all participants for SARS-CoV-2 testing. Throughout the study period, stool samples were collected from participants presenting with two or more GI symptoms, and eschar swabs were collected from those presenting with an eschar between 0.5 cm and 3 cm in size.

### Vector-borne disease real-time polymerase chain reaction testing.

Whole blood samples from all participants were preserved in Zymo 2X DNA/RNA shield (Zymo Research Inc., Irvine, CA) in a 1:1 ratio and then extracted using a modified protocol of Zymo Research Quick-DNA/RNA Pathogen Miniprep extraction kit (Zymo Research Inc.). Eluted nucleic acid samples were tested on two multiplex real-time polymerase chain reaction (RT-PCR) panels for ZIKV, WNV, pan-DENV, CHIKV, pan-*Rickettsia*, *Trypanosoma cruzi* (*T. cruzi*), and pan-*Plasmodium* (Supplemental Tables 1 and 2).^18^^–^^23^ Specimens that were pan-DENV positive by RT-PCR were serotyped using RT-PCR.^24^ After it was determined that pan-DENV PCR exhibited low sensitivity for DENV-4 serotypes circulating in Central America, all specimens were retested using a multiplex DENV1–4 serotyping assay. Eschar swabs were extracted using a modified protocol for the Qiagen DNeasy Blood & Tissue Kit (Qiagen, Hilden, Germany) and tested for pan-*Rickettsia* via RT-PCR.

### Respiratory pathogen testing.

Nasopharyngeal swabs from patients with two or more respiratory symptoms were tested on the BioFire® FilmArray Torch 2 System Respiratory 2 (RP2) panel according to the manufacturer’s instructions (BioFire Diagnostics, Salt Lake City, UT) until May 7, 2021, after which the BioFire Respiratory 2.1 panel with SARS-CoV-2 was implemented (BioFire Diagnostics; see product disclaimer). Both panels test for 22 viral and bacterial pathogens, including adenovirus; coronaviruses HKU1, NL63, 229E, and OC43; human metapneumovirus; human rhinovirus/enterovirus; influenzas A, A/H1, A/H1-2009, A/H3, and B; parainfluenza virus (1, 2, 3, and 4); RSV; SARS-CoV-2; *Bordetella parapertussis*; *Bordetella pertussis*; *Chlamydia pneumoniae*; and *Mycoplasma pneumoniae* (Supplemental Table 1). Beginning August 8, 2020, NP swabs from patients with respiratory symptoms were first screened via RT-PCR for SARS-CoV-2 using Charité – Universitätsmedizin Berlin Institute of Virology (Berlin, Germany) protocols and TaqPath 1-Step Multistep Master Mix (Applied Biosystems, Waltham, MA).^25^ If SARS-CoV-2 test results were negative, then samples from patients with two or more respiratory symptoms were tested on the BioFire respiratory panel. Because of resource constraints that occurred toward the end of the surveillance period, 67 COVID-19-negative subjects with fever and two or more respiratory symptoms were unable to be tested on the BioFire RP2 panel. As an alternative approach, samples were tested for influenza and RSV using the Xpert® Xpress CoV-2/Flu/RSV plus* (Cepheid United States, Sunnyvale, CA; see product disclaimer).

### Gastrointestinal pathogen testing.

Stool samples were tested using the BioFire FilmArray Torch System (BioFire Diagnostics) on the GI panel, according to the manufacturer’s instructions. The GI panel is used to test for 22 pathogens, including *Campylobacter jejuni, Campylobacter coli, Campylobacter upsaliensis*, *Clostridium difficile* (*C. difficile*; toxin A/B), *Plesiomonas shigelloides*, *Salmonella*, *Yersinia enterocolitica*, *Vibrio parahaemolyticus, Vibrio vulnificus, Vibrio cholerae* (*V. cholerae*), enteroaggregative *Escherichia coli* (*E. coli*; EAEC), enteropathogenic *E. coli* (EPEC), enterotoxigenic *E. coli* (ETEC) lt/st, Shiga-like toxin-producing *E. coli* (STEC) stx1/stx2, *E. coli* O157, *Shigella*/enteroinvasive *E. coli* (EIEC), *Cryptosporidium*, *Cyclospora cayetanensis* (*C. cayetanensis*), *Entamoeba histolytica* (*E. histolytica*), *Giardia lamblia*, adenovirus F40/41, astrovirus, norovirus genotypes GI and GII (GI/GII), rotavirus A, and sapovirus I, II, IV, and V (Supplemental Table 1).

### Data handling, storage, and analysis.

Demographic, epidemiological, clinical, and test result data were entered into a secure Research Electronic Data Capture database (REDCap, Vanderbilt University, Nashville, TN). Simultaneously, these results were entered into the Belize Healthcare Information System (BHIS), a nationwide electronic medical record system, so that participants’ healthcare providers would be informed of them. All positive pathogen results were communicated to the provider for clinical decision-making, treatment, and patient management. Data were analyzed using Stata v. 17.0 (StataCorp, College Station, TX), and data figures were generated using Prism 10 (Graphpad, San Diego, CA). Geospatial mapping was performed using ArcGIS Pro v. 3.0 (ESRI, Redlands, CA).

## RESULTS

### Enrollment and demographics.

**Figure 3.**
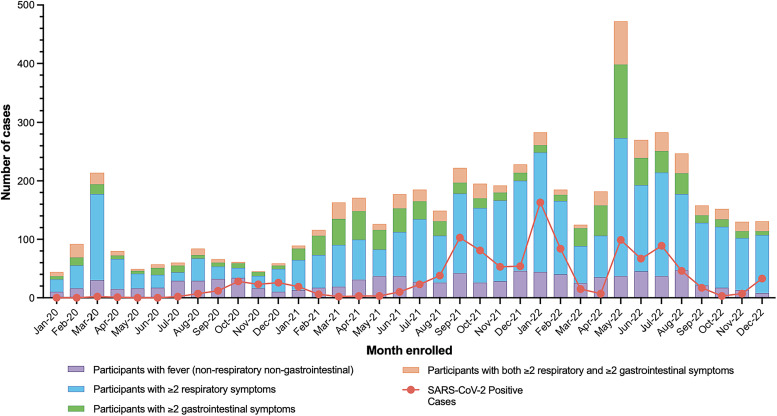
Epidemiological curve of all enrolled participants with acute febrile illness by month of enrollment date and disease presentation.

Between January 16, 2020 and December 31, 2022, 5,643 participants were enrolled in the Belize AFI surveillance network. The highest number of participants was enrolled in Cayo District (28.5%; *n* = 1,607), followed by Toledo (19.4%; *n* = 1,095) and Belize Districts (17.3%; *n* = 979) (Table 1). In the first year, the highest monthly enrollment occurred in March 2020 (*n* = 217), which was followed by a steep decline in subsequent months, coinciding with COVID-19 pandemic lockdowns and curfews initiated on March 21, 2020 (Figure 3). Enrollment steadily increased in late 2020 as cases of SARS-CoV-2 emerged across the country.^26^^,^^27^ In February 2021, enrollment was strengthened when AFI surveillance field officers were hired and placed at participating healthcare facilities to assist with enrollment. Enrollment numbers increased in September 2021, January 2022, and May 2022, coinciding with waves of increased SARS-CoV-2 cases. Throughout the 3 years of AFI surveillance in Belize, participants with two or more respiratory symptoms made up 50% or more of participants enrolled monthly, whereas those with two or more GI symptoms comprised 4.6–28.3% of the total participants enrolled per month.

**Table 1:** Acute febrile illness enrollment by location, January 16, 2020 to December 31, 2022, Belize

	*n*	%
District Health Facility	*N* = 5,643	
Cayo District		
San Ignacio Community Hospital	1,019	18.1
Western Regional Hospital	588	10.4
Toledo District		
Punta Gorda Community Hospital	1,095	19.4
Belize District		
Matron Roberts Polyclinic II	127	2.3
Karl Heusner Memorial Hospital	31	0.6
Cleopatra White Polyclinic II	736	13.0
San Pedro Polyclinic II	85	1.5
Stann Creek District		
Independence Polyclinic II	139	2.5
Southern Regional Hospital	512	9.1
Orange Walk District		
Northern Regional Hospital	841	14.9
Corozal District		
Corozal Community Hospital	243	4.3
Other health facilities		
Other health facility*	227	4.0
Belize Healthcare Partners Limited (Belize District)	109/227	48.0
Central Medical Laboratory (Belize District)	36/227	15.9
Isabella Palma Polyclinic II (Toledo District)	23/227	10.1
San Antonio Polyclinic II (Toledo District)	27/227	11.9

* Other health facility includes the Belize Gastrointestinal Clinic (Belize Specialty Hospital), Belize Healthcare Partners Limited, Belize Medical Associates, Big Falls Community Health Center, Central Health Region, Central Medical Laboratory, Chunox Polyclinic, Department of Corrections, (Kolbe Foundation), Isabella Palma Polyclinic II, Placencia Health Center, San Antonio Polyclinic II, and Santa Ana Health Center.

The median participant age was 26.7 years (range 61 days to 97.7 years), and almost one-third of the participants (*n* = 1,642; 29.1%) were 15 years of age or younger. Slightly more than half of the participants (*n* = 2,970; 52.6%) were female; 70 (2.3%) were pregnant. The most commonly reported occupations were professional/technical/managerial (*n* = 403; 7.1%) and student (*n* = 258; 4.6%); 1,094 (19.4%) participants responded as not applicable, with the majority of those being <5 years old (*n* = 1,037; 94.8%; Table 2). A total of 53 participants reported two or more occupations. Occupation was missing or not reported for 4,109 participants, including 2,581 adults (62.8%) and 1,030 children under 5 years of age (25.1%).

**Table 2:** Demographics and epidemiologic data of enrolled participants, Acute Febrile Illness Surveillance Study, Belize, 2020–2022

Variable	*n* (*N* = 5,643)	% (*N* = 5,643)	% of *n*
Female	2,970	52.6	–
Pregnant	70/2,970	2.3	–
Age (median [range])	26.7 years	61 days to 97.7 years	–
<5 years	958	17.0	–
5 to 20 years	1,084	19.2	–
21 to 40 years	2,552	45.2	–
41 to 65 years	1,090	19.3	–
>65 years	259	4.6	–
Occupation (*n* = 1,534)			Of 1,534
Not reported	4,109	72.8	–
Not applicable	1,094	19.4	71.3
Professional, technical, managerial	403	7.1	26.3
Student	258	4.6	16.8
Sales and services	248	4.4	16.2
Unemployed/retired	196	3.5	12.8
Domestic services	125	2.2	8.1
Skilled manual	88	1.6	5.7
Agriculture, forestry, fishery	76	1.4	5.0
Armed forces	55	1.0	3.6
Clerical support	52	0.9	3.4
Unskilled manual	52	0.9	3.4
Other occupation*	40	0.7	2.6
Two or more occupations	53	0.9	3.5
Recent travel (within 30 days; *n* = 5,597)	547	9.8	–
Exposure to ill individuals^†^ (*n* = 5,575)	1,209	21.7	–
Exposure to vectors^†^ (*n* = 5,593)	3,630	64.9	Of 3,630
Mosquito	3,523	63.0	97.1
Sand fly	1,211	21.7	33.4
Tick	197	3.5	5.4
Flea	195	3.5	5.4
Triatomine	50	0.9	1.4
Other vector	3	0.1	0.1
Exposure to animals^†^ (*n* = 1,736)	903	52.0	of 903
Dogs	828	47.7	91.7
Cats	300	17.3	33.2
Chickens or other poultry	198	11.4	21.9
Rodents	39	2.2	4.3
Pigs	37	2.1	4.1
Small ruminants (sheep and goats)	29	1.7	3.2
Cows	27	1.6	3.0
Bats	11	0.6	1.2
Horses	6	0.3	0.7
Other animal(s)^‡^	16	0.9	1.8

* Other occupation was unspecified.

^†^ Exposure occurred within 2 weeks of presentation and enrollment. Exposure to animals was collected until November 1, 2021 and then removed from the case investigation form and moved to a follow-up questionnaire.

^‡^ Other animals include birds (2), bush meat (3), deer (1), opossums (1), parrot (2), pigeons (1), and rabbits (5).

Only 9.8% of participants reported travel history within 30 days of presentation, and nearly one-quarter (21.7%) of participants reported contact with an ill individual within the previous 2 weeks (Table 2). More than half of participants (64.9%) reported exposure to vectors within the previous 2 weeks, with most reporting mosquitoes (97.1%). Other reported vectors included sand flies (33.4%), ticks (5.4%), fleas (5.4%), and triatomines (1.4%; Table 2). Approximately half of the participants (52.0%) reported animal exposure within 2 weeks of presentation; the most common contacts were dogs (91.7%), cats (33.2%), and chickens or other poultry (21.9%; Table 2).

### Symptoms.

The median temperature of all participants was 37.9°C (with a range of 35.0–42.2°C). The median time from the onset of symptoms to enrollment was 2 days. The most commonly reported symptoms included fever (77.6%), headache (48.5%), fatigue (36.0%), chills (32.0%), muscle pain or aches (25.1%), arthralgia (20.2%), and loss of appetite (18.6%; Table 3). More than half of the participants (*n* = 3,725; 66.0%) reported two or more respiratory symptoms, whereas one-quarter of the participants (*n* = 1,455; 25.8%) reported two or more GI symptoms. The most commonly reported respiratory symptoms were cough (59.6%), runny nose or nasal congestion (49.7%), sore throat (42.3%), sneezing (27.6%), shortness of breath (21.1%), chest pain (15.9%), loss of smell or taste (7.2%), and wheezing (6.8%; Table 3). The most commonly reported GI symptoms included diarrhea (22.8%), vomiting (19.9%), abdominal or stomach pain (18.4%), nausea (16.5%), and upset stomach (10.0%; Table 3).

**Table 3:** Acute febrile illness self-reported symptoms of enrolled cases

Self-Reported Symptoms	Total (*N* = 5,643)	%
General symptoms		
Fever	4,379	77.6
Headache	2,739	48.5
Fatigue	2,030	36.0
Chills	1,807	32.0
Muscle pains and aches	1,415	25.1
Arthralgia	1,138	20.2
No appetite	1,049	18.6
Retro orbital pain	597	10.6
Unusual sweating	318	5.6
Rash	195	3.5
Dizzy	170	3.0
Stiff neck	136	2.4
Conjunctivitis	122	2.2
Weight loss	108	1.9
Swelling	57	1.0
Dysuria	48	0.9
Eschar	43	0.8
Swollen lymph nodes	42	0.7
Other general symptom(s)*	37	0.7
Jaundice	29	0.5
Convulsions	28	0.5
Flush	26	0.5
Unusual bleeding	18	0.3
Paralysis	7	0.1
Chagoma or Romaña sign	5	0.1
Respiratory symptoms		
Reported two or more respiratory symptoms	3,725	66.0
Cough	3,365	59.6
Runny nose/nasal congestion	2,804	49.7
Sore throat/painful swallowing	2,387	42.3
Sneezing	1,559	27.6
Shortness of breath	1,190	21.1
Chest pain	895	15.9
Loss of smell/taste	404	7.2
Wheezing	384	6.8
Other respiratory symptom(s)^†^	30	0.5
GI symptoms		
Reported two or more GI symptoms	1,455	25.8
Diarrhea	1,286	22.8
Vomiting	1,124	19.9
Abdominal or stomach pain	1,040	18.4
Nausea	932	16.5
Upset stomach	564	10.0
Black stool or blood in stool	67	1.2
Other GI symptom(s)^‡^	13	0.2

GI = gastrointestinal.

* Other general symptoms include blisters, deep vein thrombosis, dysphagia, frequent urination, heart palpitations, insomnia, lump on the back of the neck, memory loss, mouth sores, nose bleeding, semi-consciousness, skin lesions, spots on hands and feet, tachycardia, and trismus.

^†^ Other respiratory symptoms include coughing up blood, desaturation, earache, hiccups, and sinusitis.

^‡^ Other GI symptoms include constipation, gas, and vomiting blood.

### Vector-borne pathogen detection.

**Figure 4.**
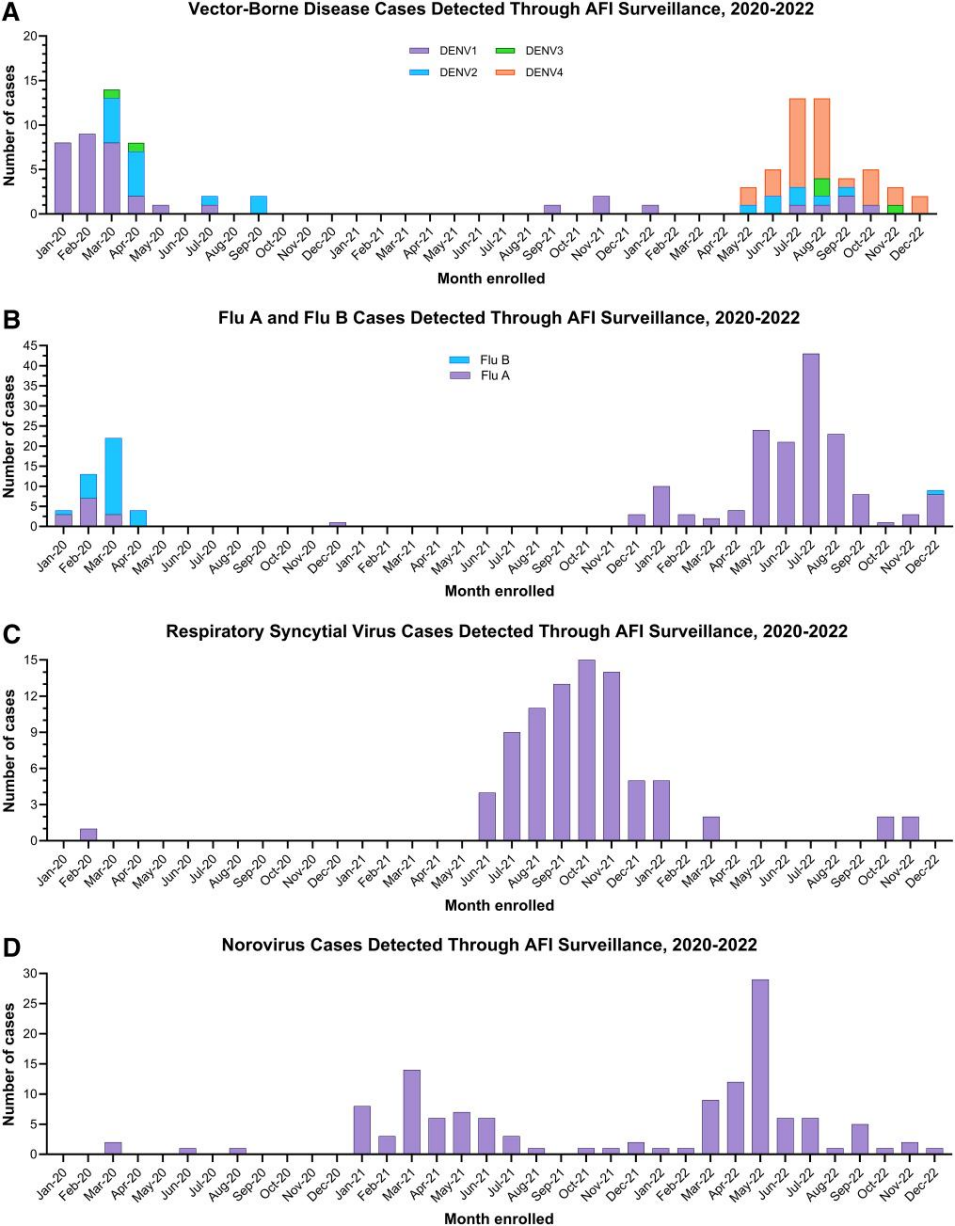
Number of disease cases detected via acute febrile surveillance in Belize (2020–2022) for vector-borne diseases, flu, respiratory syncytial virus, and norovirus.

Almost all participants (*n* = 5,488; 97.3%) had WB samples available for testing on the VBD RT-PCR panels, with 1.7% of participants (91/5,488) testing positive. The most common VBD detected was DENV, with 99 DENV-positive cases, including 44 DENV-positive cases in 2020 (5.2% of the 845 tested), three cases in 2021 (0.1% of the 2,005 tested), and 52 cases in 2022 (2.0% of the 2,588 tested). One *T. cruzi* case was detected in 2020. All participants were negative for ZIKV, WNV, CHIKV, pan-*Rickettsia*, and pan-*Plasmodium* across all 3 years (Table 4). Eschar swabs were collected from 26 participants (0.47%), and none were found to be positive for *Rickettsia* spp. The majority of the VBDs found in 2020 (26/39; 67%), including one *T. cruzi* case, were detected during the first quarter of the year, with a dramatic drop in identified cases after the onset of the COVID-19 pandemic and nationwide lockdowns beginning in late March 2020. In 2020, the serotypes detected included DENV-1 (*n* = 29), DENV-2 (*n* = 13), and DENV-3 (*n* = 2). In 2022, DENV cases were detected between May and December, with a peak in July and August (Figure 4A). All four serotypes were detected; however, most participants were positive for DENV-4 (33/52; 63%). Those who were DENV-positive had two times higher odds of reporting mosquito exposures in the 2 weeks before illness onset compared with those who were DENV-negative (77% versus 62%; odds ratio = 2.0; 95% confidence interval = 1.3 to 3.2; *P* = 0.0032).

**Table 4:** Vector-borne disease infections detected via multiplex real-time polymerase chain reaction testing, Acute Febrile Illness Surveillance Study, Belize, 2020–2022

Vector-Borne Disease Infection	Total Tested, *N* = 5,488		2020, *n* = 845		2021, *n* = 2,005		2022, *n* = 2,588	
	Number of Positive Tests	%	Number of Positive Tests	%	Number of Positive Tests	%	Number of Positive Tests	%
ZIKV	0	0	0	0	0	0	0	0
WNV	0	0	0	0	0	0	0	0
DENV (all serotypes)	99	1.8	44	5.2	3	0.1	52	2.0
DENV-1	41	0.7	29	3.4	3	0.1	9	0.3
DENV-2	20	0.4	13	1.5	0	0	7	0.3
DENV-3	5	0.09	2	0.2	0	0	3	0.1
DENV-4	33	0.6	0	0	0	0	33	1.3
CHIKV	0	0	0	0	0	0	0	0
*Pan*-*Rickettsia*	0	0	0	0	0	0	0	0
*Trypanosoma cruzi*	1	0.02	1	0.1	0	0	0	0
*Pan*-*Plasmodium*	0	0	0	0	0	0	0	0

CHIKV = chikungunya virus; DENV = dengue virus; WNV = West Nile virus; ZIKV = Zika virus.

### Respiratory pathogen detection.

**Figure 5.**
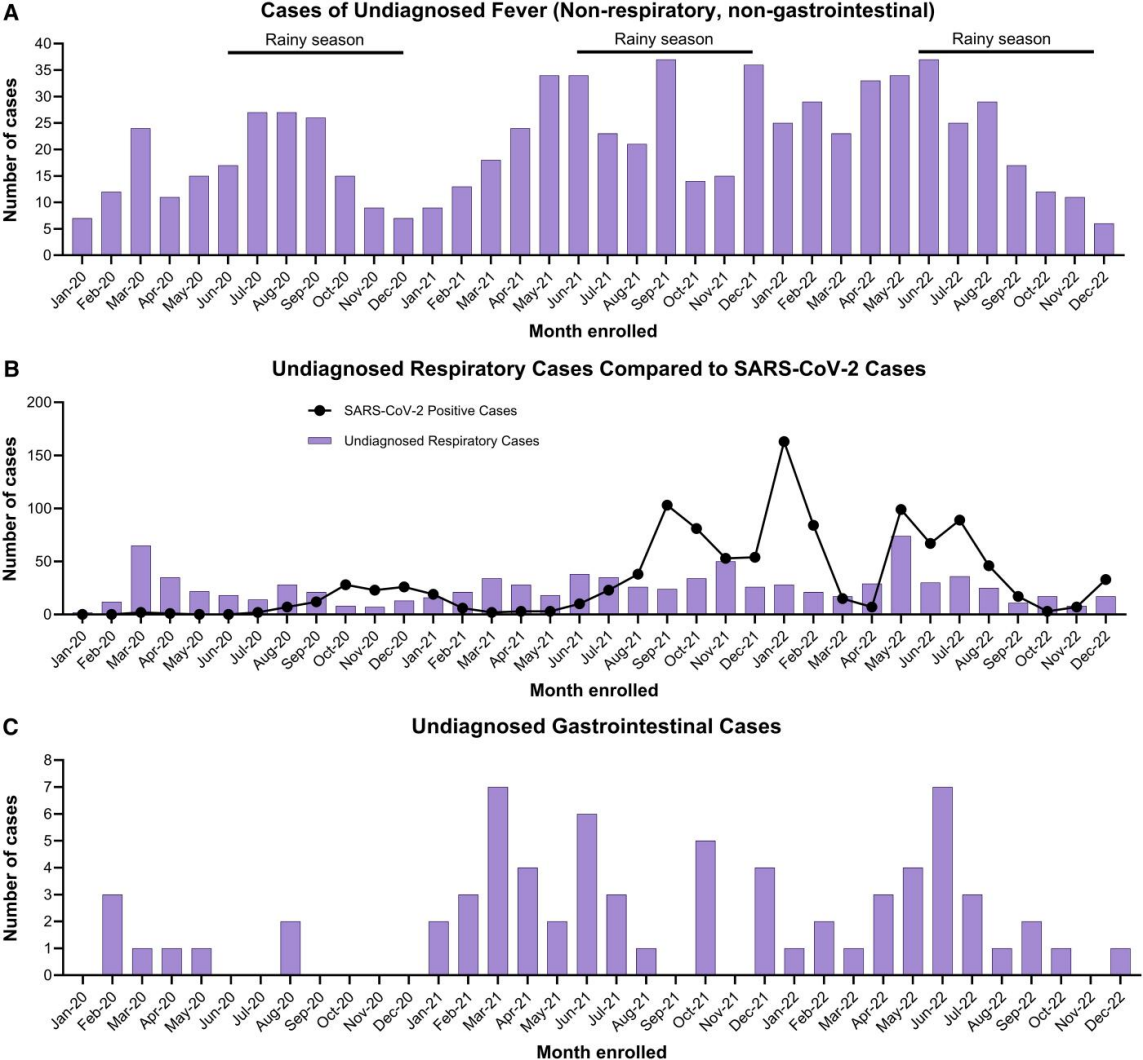
Undiagnosed cases in the Acute Febrile Illness Surveillance Study in Belize between 2020 and 2022 among participants with fever (non-respiratory, non-gastrointestinal), two or more respiratory symptoms, or two or more gastrointestinal symptoms.

Most participants (*n* = 4,516; 80.0%) had NP swabs collected for SARS-CoV-2 PCR testing; 1,127 (25.0%) tested positive (Table 5). Of those tested for SARS-CoV-2, 3,161 (72.9%) presented with two or more respiratory symptoms, of whom 921 (29.1%) were positive. Among participants without two or more respiratory symptoms, 14.5% (171/1,178) were positive for SARS-CoV-2. The incidence of SARS-CoV-2 infection among participants was highest in 2022, with 630 (29.7%) testing positive (Table 5); one-quarter of these cases (*n* = 163; 25.9%) were detected during a surge in January 2022 (Figure 5B). Those who tested positive for SARS-CoV-2 but lacked two or more respiratory symptoms were significantly more likely to report two or more GI symptoms (37/171 [21.6%] versus 120/921 [13.0%]; *P* <0.01).

**Table 5:** Respiratory infections detected among study participants

	Total		2020		2021		2022	
Respiratory Testing* Performed	Number of Positive Results/Number of Subjects Tested	%	Number of Positive Results/Number of Subjects Tested	%	Number of Positive Results/Number of Subjects Tested	%	Number of Positive Results/Number of Subjects Tested	%
SARS-CoV-2 RT-PCR	1,127/4,516	25.0	102/632	16.1	395/1,766	22.4	630/2,118	29.7
Human rhinovirus/enterovirus	586/2,518	23.3	111/635	17.5	334/1,108	30.1	141/775	18.2
Influenza A/H3	149/2,518	5.9	0/635	0.0	3/1,108	0.3	146/775	18.8
Coronavirus OC43	94/2,518	3.7	0/635	0.0	91/1,108	8.2	3/775	0.4
Human metapneumovirus	91/2,518	3.6	14/635	2.2	34/1,108	3.1	43/775	5.5
Respiratory syncytial virus	89/2,587	3.4	1/635	0.2	71/1,108	6.4	17/844	2.0
Adenovirus	71/2,518	2.8	21/635	3.3	30/1,108	2.7	20/775	2.6
Parainfluenza virus 3	66/2,518	2.6	1/635	0.2	15/1,108	1.4	50/775	6.5
Coronavirus 229E	39/2,518	1.5	9/635	1.4	2/1,108	0.2	28/775	3.6
Influenza A	43/2,591	1.7	1/635	0.2	0/1,108	0.0	42/848	5.0
Influenza B	31/2,591	1.2	30/635	4.7	0/1,108	0.0	1/848	0.1
Parainfluenza virus 1	28/2,518	1.1	18/635	2.8	5/1,108	0.5	5/775	0.6
Coronavirus NL63	24/2,518	1.0	2/635	0.3	21/1,108	1.9	1/775	0.1
Parainfluenza virus 4	19/2,518	0.8	6/635	0.9	2/1,108	0.2	11/775	1.4
Coronavirus HKU1	15/2,518	0.6	8/635	1.3	1/1,108	0.1	6/775	0.8
Influenza A/H1-2009	13/2,518	0.5	13/635	2.0	0/1,108	0.0	0/775	0.0
Parainfluenza virus 2	11/2,518	0.4	6/635	0.9	2/1,108	0.2	3/775	0.4
*Chlamydia pneumoniae*	4/2,518	0.2	3/635	0.5	1/1,108	0.1	0/775	0.0
*Mycoplasma pneumoniae*	3/2,518	0.1	3/635	0.5	0/1,108	0.0	0/775	0.0
Influenza A/H1	1/2,518	0.04	0/635	0.0	0/1,108	0.0	1/775	0.0
*Bordetella parapertussis*	0/2,518	0.0	0/635	0.0	0/1,108	0.0	0/775	0.0
*Bordetella pertussis*	0/2,518	0.0	0/635	0.0	0/1,108	0.0	0/775	0.0
One or more pathogens detected on BioFire® panel	1,196/2,518	47.5	212/635	33.4	556/1,108	50.2	428/775	55.2

RT-PCR = real-time polymerase chain reaction; SARS-CoV-2 = severe acute respiratory syndrome coronavirus 2.

* Unless otherwise noted, all testing was conducted on the BioFire panel, with the exception of SARS-CoV-2 testing, which was completed using RT-PCR, Xpert® Xpress CoV-2/Flu/RSV *plus*, or BioFire.

Of the 4,380 participants with an NP swab collected, 2,518 (44.6%) were tested on the BioFire respiratory panel; almost half (*n* = 1,200; 47.7%) had at least one positive result (Table 5). Over the 3-year surveillance period, the most common respiratory pathogen detected on the BioFire respiratory panel was human rhinovirus/enterovirus (23.3%), followed by influenza A/H3 (5.9%), coronavirus OC43 (3.7%), human metapneumovirus (3.6%), and RSV (3.4%; Table 5). Of the 67 participants in 2022 who were tested on Xpert Xpress CoV-2/Flu/RSV *plus*, 11 (16.4%) were positive for influenza A, none were positive for influenza B, and six (9.0%) were positive for RSV. Influenza cases were detected during the first quarter of 2020 before lockdowns were conducted (Figure 4B), followed by an absence of detected cases for 19 months, except for a single positive case in December 2020. Most positive cases in 2020 were influenza B, whereas all positive cases in 2022 were influenza A. Conversely, most RSV cases were detected in 2021, with a peak in October 2021 (Figure 4B).

### Gastrointestinal pathogen detection.

Stool was collected from 12.1% of participants (*n* = 680) and tested on the BioFire GI panel; most reported two or more GI symptoms (94%; 642/680). The majority (*n* = 575; 84.6%) yielded at least one positive result. Diarrheagenic *E. coli* pathogens (EPEC, EAEC, ETEC, EIEC, STEC, and *E. coli* 0,157) were common, with 69.4% (472/680) of participants having at least one strain detected. The most common diarrheagenic *E. coli* strains included EPEC (46.3%), EAEC (30.0%), EIEC (17.1%), and ETEC (16.2%; Table 6). *Campylobacter* bacterial infections were also prevalent (14.7%). Twelve participants tested positive for *Vibrio* species, including one child who tested positive for *V. cholerae* in 2021 and seven other participants who tested positive for *V. cholerae* in 2022. Three of the cholera patients in 2022 yielded a positive culture, and PCR and sequencing confirmed non-01/0139 (non-epidemic) strains.

**Table 6:** Gastrointestinal infections detected among study participants with acute febrile illness

	Total Tested, *N* = 680		2020, *n* = 95		2021, *n* = 256		2022, *n* = 329	
GI pathogen detected on BioFire® panel	Number of Positive Results	%	Number of Positive Results	%	Number of Positive Results	%	Number of Positive Results	%
*Enteropathogenic E. coli*	315	46.3	38	40.0	112	43.8	165	50.2
*Enteroaggregative E. coli*	204	30.0	24	25.3	70	27.3	110	33.4
Norovirus GI/GII	130	19.1	4	4.2	52	20.3	74	22.5
*Shigella/Enteroinvasive E. coli*	116	17.1	25	26.3	34	13.3	57	17.3
*Enterotoxigenic E. coli* lt/st	110	16.2	16	16.8	40	15.6	54	16.4
*Campylobacter*	100	14.7	12	12.6	35	13.7	53	16.1
Rotavirus A	70	10.3	1	1.1	13	5.1	56	17.0
*Giardia lamblia*	64	9.4	8	8.4	15	5.9	41	12.5
*Salmonella*	33	4.9	9	9.5	10	3.9	14	4.3
*Shiga-like toxin-producing E. coli stx1/stx2*	28	4.1	5	5.3	10	3.9	13	4.0
*Plesiomonas shigelloides*	27	4.0	2	2.1	13	5.1	12	3.6
Adenovirus F40/41	23	3.4	4	4.2	7	2.7	12	3.6
*Clostridium difficile*	20	2.9	1	1.1	14	5.5	5	1.5
*Cryptosporidium*	17	2.5	1	1.1	5	2.0	11	3.3
Astrovirus	14	2.1	1	1.1	7	2.7	6	1.8
Sapovirus	14	2.1	2	2.1	2	0.8	10	3.0
*Vibrio (parahaemolyticus, vulnificus, cholerae)*	12	1.8	2	2.1	4	1.6	6	1.8
*E. coli O157*	9	1.3	3	3.2	2	0.8	4	1.2
*Vibrio cholerae*	8	1.2	0	0.0	1	0.4	7	2.1
*Cyclospora cayetanensis*	2	0.3	0	0.0	1	0.4	1	0.3
*Entamoeba histolytica*	1	0.1	0	0.0	0	0.0	1	0.3
*Yersinia enterocolitica*	0	0.0	0	0.0	0	0.0	0	0.0
Had one or more pathogens detected on BioFire panel	575	84.6	76	80.0	206	80.5	293	89.1

*E. coli* = *Escherichia coli*; GI = gastrointestinal; GI/GII = genotypes I and II.

Norovirus GI/GII was the most common viral GI pathogen detected (19.1%). Cases increased in January 2021 and continued through half the year, followed by a second wave beginning in March 2022, which peaked in May 2022 (Figure 4D). Rotavirus A was the second most detected viral GI pathogen (10.3%), with more cases detected in 2022 compared with the previous 2 years (Table 6). The majority (55/70; 79%) of positive rotavirus cases were among children 18 years of age and younger.

Although parasitic GI pathogens were less common, 9.4% of participants tested positive for *Giardia*. *Cryptosporidium* was the second most common parasitic infection, with 17 (2.5%) cases detected, whereas <1% of participants tested positive for *C. cayetanensis* and *E. histolytica*.

### Unknown pathogens.

Approximately half of the participants (49.1%; 2,772/5,643) did not have a pathogen identified on testing. A higher percentage of febrile participants without respiratory or GI symptoms (77.8%; 756/972) were undiagnosed compared with those who reported two or more respiratory symptoms (41.3%; 908/2,197) or two or more GI symptoms (11.1%; 71/642). Cases of undiagnosed fever (non-respiratory, non-GI) appeared to have similar waves in all 3 years (Figure 5A), with increases in cases observed between May and September, which somewhat coincided with the rainy season (June to December). Increases in undiagnosed respiratory cases were observed at the beginning of the surveillance period (February to May 2020) and toward the end of the surveillance period (August to December 2022; Figure 5B). Undiagnosed GI (Figure 5C) case peaks mirrored increases in norovirus GI/GII cases (Figure 4D), with peaks between January and July 2021, as well as between March and September 2022.

### Coinfections.

Coinfections were fairly common, with 601 participants (10.7%) testing positive for two or more pathogens. Coinfections were especially prevalent among those tested on the GI panel, with more than half of the participants (57.5%) having two or more pathogens detected; the most common coinfections were EPEC with EAEC, followed by EPEC with norovirus. Four or more pathogens were detected in 64 participants (2.1%). One 2.5-year-old child had eight pathogens detected, including adenovirus, coronavirus NL63, *C. difficile*, EAEC, EPEC, ETEC, EIEC, and *Giardia*. Fourteen participants were coinfected with a VBD and a respiratory pathogen (0.2%), nine participants were coinfected with a VBD and a GI pathogen (0.2%), 94 participants (1.7%) had a respiratory and GI coinfection, and two (0.04%) were coinfected with a VBD and respiratory and GI pathogens. Enterotoxigenic *E. coli* and human rhinovirus/enterovirus were most often detected in coinfection cases.

## DISCUSSION

Before this AFI surveillance network was implemented, Belize had limited infrastructure and resources for detecting vector-borne, respiratory, and GI pathogens. Through the AFI surveillance program, Belize’s local capacity to diagnose 54 unique vector-borne, respiratory, and GI infections was markedly expanded. As a result, the first known case of acute Chagas disease was diagnosed in a 7-year-old child, leading to swift treatment and vector control measures.^28^ Along with VBD RT-PCR testing, the BioFire FilmArray Torch System was instrumental in expanding Belize’s diagnostic capabilities, and the GI panel enabled the detection of a small outbreak of non-epidemic *V. cholerae*, facilitating rapid response and control measures, thereby limiting its spread. Although BioFire is a cost-prohibitive platform for most LMICs, the comprehensive surveillance data produced in real-time provided an opportunity to define the key pathogens for future targeted testing using more sustainable approaches. Through comprehensive testing platforms, pathogens were successfully detected in half of the study participants over the 3-year surveillance period.

With the onset of the COVID-19 pandemic, testing was quickly expanded to include SARS-CoV-2. Unsurprisingly, SARS-CoV-2 was the most diagnosed pathogen among AFI cases, with one in four participants testing positive. The high incidence was partially driven by the decision to implement universal testing for SARS-CoV-2 regardless of presenting symptoms, as 9.2% of those without two or more respiratory symptoms tested positive for SARS-CoV-2 by PCR. The proportionally high rates of SARS-CoV-2 compared with other respiratory pathogens could also reflect changes in healthcare-seeking behaviors for non-COVID-19 illnesses during the pandemic. Those with mild symptoms may have elected not to seek care because of their perceived risk of being exposed to SARS-CoV-2 in a healthcare setting.^29^ Alternatively, non-pharmaceutical measures such as masks and quarantine measures likely played a role in limiting the transmission of other communicable diseases, especially in the first 2 years of the pandemic.^30^ This was likely the case with DENV because a sudden drop in cases was observed in 2020 at the onset of travel restrictions and quarantine measures, followed by only 3 detected cases in 2021, and the resurgence of cases in 2022 when restrictions were fully lifted. Ultimately, the SARS-CoV-2 pandemic exemplifies the importance of an adaptable AFI surveillance network to detect emerging infections in vulnerable and under-resourced settings.

Healthcare workers in resource-limited settings often empirically base their diagnoses on presenting symptoms and their knowledge of the local epidemiology of a disease.^31^^,^^32^ Through this enhanced AFI surveillance, surveillance findings could be communicated in real-time via automated data analytics presented in an online “story map” ArcGIS dashboard and weekly emails to stakeholders. These results not only informed timely public health responses but also provided healthcare providers with an evidence-based approach to patient assessment and care. Furthermore, the rapid turnaround time in communicating results enabled targeted therapies and infection control measures.^33^ Belize employs a country-wide electronic medical record system (BHIS), which has greatly improved communication of results to healthcare providers and public health officials.

Because of difficulties in collecting stool samples at the time of presentation, only approximately half of the participants (44.1%) who presented with GI symptoms could be tested on the GI pathogen panel. Nevertheless, the prevalence of GI pathogens in the population during the study period was successfully examined, with a high percentage (84.6%) of those tested having one or more pathogens identified. In a study on the patterns of illness among travelers returning to the United States, researchers reported acute diarrhea as one of the most commonly reported symptoms when returning from Central America.^34^ In another study, 63% of students who spent 5 weeks in Mexico developed diarrhea, with the majority of infections caused by ETEC and EAEC, which were also highly prevalent in the present study’s AFI population. Interestingly, almost half of the cases (42.7%) in the current study were positive for EPEC, which is higher than previously reported in Costa Rica (24%) and Guatemala (8% and 12% for hospitalized and outpatient diarrheal illness cases, respectively).^35^^–^^37^

Norovirus was also a common GI pathogen detected in the present study, which is unsurprising, given its higher prevalence in LMICs compared with upper-middle- and high-middle-income countries.^38^ Rotavirus was the second most common viral GI pathogen, with the majority of cases presenting in children. Worldwide, rotavirus accounts for 37% of diarrhea-related deaths in children, with LMICs accounting for 92% of those deaths.^39^ In Belize, rotavirus vaccination is not included as part of the routine immunization schedule.^39^^,^^40^

Although the study panel of 54 pathogens was robust, it does not cover all potential disease-causing organisms in the region. The four arboviruses were chosen on the basis of their epidemiology in the Americas, but others in the flavivirus and alphavirus genera could be considered in future testing. In a syndromic surveillance study conducted in Ecuador, Peru, Bolivia, and Paraguay, additional viruses in the alphavirus and bunyavirus genera were included in testing, such as Venezuelan equine encephalitis virus, Mayaro virus, Oropouche virus, and Group C viruses.^41^ Much like in the present study, DENV was the most detected vector-borne virus.^41^ A large AFI surveillance study conducted in Puerto Rico from 2012 to 2015 revealed the presence of DENV 1–4, CHIKV, enterovirus, *Leptospira* spp., *Burkholderia pseudomallei*, and various respiratory pathogens.^42^ In the Puerto Rico study, the most common arbovirus was CHIKV, which reflected the widespread epidemic that occurred in 2014.^43^ In addition to the arboviruses listed above, other vector-borne and zoonotic pathogens to consider for inclusion are *Leishmania* spp., *Ehrlichia* spp., *Borrelia* spp., *Leptospira* spp., and *Brucella* spp. Although the BioFire panels are comprehensive, future respiratory and GI testing could include targeted diagnostics and sequencing to identify other pathogens in those who tested negative.

The pan-*Rickettsia* PCR test was designed to detect both spotted fever group and typhus group *Rickettsia* in both WB and eschar samples; however, the test yielded no positive results. It is possible that patients did not have high enough levels of bacteremia in their WB samples to be detectable via PCR, and other pathogens can cause eschar formation. *Rickettsia*-positive cases were expected in the present study because a previous study revealed that 54.0% of tested individuals were seropositive for spotted fever group *Rickettsia* in Belize.^44^ Future studies should be focused on the serological diagnosis of acute cases using both acute and convalescent specimens.

As with all surveillance efforts, the present study faced several limitations. One limitation is that the total number of patients with AFI who presented to each participating facility and would have been eligible for enrollment is unknown. This uncertainty limited the possibility of evaluating nonresponse bias, given that participants who enrolled may differ in important ways from those who did not enroll. Enrollment varied substantially by site and location, which could have been influenced by factors such as facility accessibility, healthcare provider participation in patient enrollment, and the presence of AFI technical staff to assist with enrollment. Surveillance system training and sensitizations were conducted at the onset of the study to provide knowledge and tools to encourage enrollment; however, staff turnover, transfers, and shortages, along with COVID-19 restrictions, may have impacted enrollment over time. Despite DENV being the predominant arbovirus in circulation in the Americas,^45^^,^^46^ this AFI surveillance system revealed relatively few cases of DENV, which may be due to physicians’ tendency to perform a dengue rapid test at the time of care rather than enroll suspected cases into the AFI surveillance system. Although enrollment was limited to those who presented within the first 7 days of symptom onset to optimize detection of pathogens using molecular diagnostics like PCR, there is the possibility of missing cases of disease if the number of circulating pathogens in the bloodstream is too low to detect. Alternatively, the detection of a pathogen may not necessarily indicate the etiology of the presenting illness, as some pathogens, particularly those on the GI panel, can occur in asymptomatic individuals. Finally, to conserve resources, BioFire respiratory panels were only run on those who first screened negative for SARS-CoV-2, which then limited the possibility of examining respiratory coinfections in SARS-CoV-2-positive patients.

## CONCLUSION

The high enrollment across multiple healthcare facilities throughout the country, along with a robust and comprehensive pathogen testing panel, enabled this AFI surveillance network to successfully detect and further characterize the epidemiology of emerging and established vector-borne, respiratory, and GI pathogens in Belize. Importantly, this surveillance network proved adaptable to the rapid implementation of SARS-CoV-2 testing, and enrollment continued despite pandemic conditions, which provided critical support to the Belize MOHW. Similarly, continuous engagement and communication among the MOHW, BCM, and the CDC contributed to effective public health outcomes. With this infrastructure in place, the most common diseases responsible for febrile, respiratory, and GI illnesses in Belize could be identified. Important discoveries were also made, including the first reported case of acute Chagas disease in Belize and eight cases of *V. cholerae*. These data can be used to streamline and prioritize diagnostic testing for known common pathogens and facilitate the sustainability of AFI surveillance in the years to come.

## Supplemental Materials

10.4269/ajtmh.25-0328 Supplemental Materials
