# Supplementary material for: Infectious Causes of Acute Febrile Illness in Belize, 2020–2022
Source: Am J Trop Med Hyg. 2025 Dec 4;114(2):224–36. doi: 10.4269/ajtmh.25-0328 (PMC12874899; doi:10.4269/ajtmh.25-0328)
Supplement: Supplemental Materials [file tpmd250328.SD1.pdf]

Supplemental Table 1. Pathogens tested as part of the AFI surveillance network study.

**Vector-Borne Pathogens (2 Multiplex RT-PCR panels)**		
**Viral**	**Bacterial**	**Parasitic**
DENV Serotype 1	*Rickettsia* spp.	*Plasmodium* spp.
DENV Serotype 2		*Trypanosoma cruzi*
DENV Serotype 3		
DENV Serotype 4		
Chikungunya (CHIKV)		
Zika (ZIKV)		
West Nile (WNV)		
**Gastrointestinal Pathogens (BioFire®)**		
**Viral**	**Bacterial**	**Parasitic**
Adenovirus F40/41	*Campylobacter jejuni*	*Cryptosporidium*
Astrovirus	*Campylobacter coli*	*Cyclospora cayetanensis*
Norovirus GI/GII	*Campylobacter upsaliensis*	*Entamoeba histolytica*
Rotavirus A	*Clostridium difficile* (toxin A/B)	*Giardia lamblia*
Sapovirus I	*Plesiomonas shigelloides*	
Sapovirus II	*Salmonella*	
Sapovirus IV	*Yersinia enterocolitica*	
Sapovirus V	*Vibrio parahaemolyticus,*	
	Vibrio vulnificus	
	Vibrio cholerae	
	Enteroaggregative E. coli (EAEC)	
	Enteropathogenic E. coli (EPEC)	
	Enterotoxigenic E. coli (ETEC) *lt/st*	
	Shiga-like toxin-producing E. coli (STEC) *stx1/stx2*	
	E. coli O157	
	Shigella/Enteroinvasive E. coli (EIEC)	
**Respiratory Pathogens (BioFire®)**		
**Viral**	**Bacterial**	**Parasitic**
Adenovirus	*Bordetella parapertussis*	N/A
Coronavirus HKU1	*Bordetella pertussis*	
Coronavirus NL63	*Chlamydia pneumoniae*	
Coronavirus 229E	*Mycoplasma pneumoniae*	
Coronavirus OC43		
Human Metapneumovirus		
Human Rhinovirus/Enterovirus		
Influenza A[a]		

Influenza A/H1		
Influenza A/H3		
Influenza A/H1-2009		
Influenza B[a]		
Parainfluenza Virus 1		
Parainfluenza Virus 2		
Parainfluenza Virus 3		
Parainfluenza Virus 4		
Respiratory Syncytial Virus[a]		
SARS-CoV-2[a,b]		

[a]Additionally tested on the Xpert® Xpress CoV-2/Flu/RSV *plus*.

[b]Additonally tested on SARS-CoV-2 PCR outside of BioFire Respiratory Panel.

Supplemental Table 2. Primers, Probes, and Positive Control Oligos

**Pathogen**	**Forward Primer (5'→3')**	**Reverse Primer (5'→3')**	**Probe (5'→3')**	**Positive Control Sequence (5'→3')***
ZIKV: M	TTGGTCATGATACTGCTGATTGC	TTGGTCATGATACTGCTGATTGC	ABY-CGGCATACAGCATCAGGTGCATAGGAG-QSY	TCAACGAGCCAAAAAGTCATATACTTGGTCATGATACTGCTGATTGCCCCGGCATACAGCATCCAACGAATCTAAAGGTGCATAGGAGTCAGCAATAGGGACTTTGTGGAAGGTATGTCAGGTGGGACTTGGGTTGAT
WNV: NS1	TGTGGACCTTAGTGTCGTGGTT	TGAGGCGTTTAGGTGCTGACT	VIC-AACAGGAGGGAATGTAC	CTGAACACTCTTTTGAAGGAGAATGGTGTGGACCTTAGTGTCGTGGTTGAGAAGCGTATCTCTACTTACATGCGTCGTTACCCTGAGGATACTCGTCCTATGGAGGATAAACAGGAGGGAATGTACAAGTCAGCACCTAAACGCCTCACCGCCACCACGGAAAAATTGGAAATT
Pan-DENV: 3' Loop	TCACTCCCTGTTGGACTTGATAGA	TTGACGAACAGAGTTAGGAACATACC	JUN-AGGTACGCGCTTCAAGTTCGGCG-QSY	AGGAGACCCCCCCGAAATAAAAAACAGCATATTGACGCTGGGAAAGACCAGAGATCCTGCTGTCTGAAGTGCAAATACCTCAGCATCATTCCAGGCACAGAACGCCAGAAAATGGAATGGTGCTGTTGAATCAACAGGTTCT
CHIKV: NSP	GARAGACCAGAGATCCTGCTGTCT	ACCATTCCATTTTCTGGCGTT	FAM-AGCATCATTCCAGGCAC	ATGTTGTTAGAAGATTTAGGGGTGGATCACTCCCTGTTGGACTTGATAGAGGCCGCTTTTGGAGAGATTTCCAGCTGTCATCTACCGAATGTTCATACCTCAGGTACGCGCTTCAAGTTCGGCGCCATGATGAAATCTGGTATGTTCCTAACTCTGTTCGTCAACACATTGCTAAACATCACCATCGCT
Pan-*Rickettsia*	AGCTTGCTTTTGGATCATTTGG	TTCCTTGCCTTTTCATACATCTAGT	ABY-CCTGCTTCTATTTGTCTTGCAGTAACACGCCA-QSY	TGCTTCAAAAGCAAAAGCAGGTACGACGCTTGCTTTTGGATCATTCGGTCTAAAATCTATAGACGGTTGGCGTGTTACTGCAAGACAAATAGAAGCAGGAAGAAAAGCTGCTACTAGATGTATGAAAAGGCAAGGAAGATTATGGATTCGTATTTTTCCGGA
Pan-*Plasmodium*	AGCTCTTTCTTGATTTCTTGG	CAGACAAATCATATTCACGAACT	6FAM-AAACGGCCATGCATCACCAT-QSY	TTTAAGACAAGAGTAGGATTGACAGATTAATAGCTCTTTCTTGATTTCTTGGATGGTGATGCATGGCCGTTTTTAGTTCGTGAATATGATTTGTCTGGTTAATTCCGATAACGAACGAGATCTTAACCTGCTAATTAGCGG
Trypanosoma cruzi	ASTCGGCTGATCGTTTTCGA	AATTCCTCCAAGCAGCGGATA	VIC-CACACACTGGACACCAA	ASTCGGCTGATCGTTTTCGAGCGGCTGCTGCACCACACGYTGTGGTCTAAATTTTTGTTTCGAATTAWGRATGRTGGGAGTCAGAGGCACTCTCTTTCACTATCTGTTTGYGTGTTCACACACTGGACGCCAAACAACCCTGAACTATCCGCTGCTTGGAGGAATT

*Positive control sequences contain inserted regions to identify the oligo from a pathogen source.
